# Sustained Inhibition of GABA-AT by OV329 Enhances Neuronal Inhibition and Prevents Development of Benzodiazepine Refractory Seizures

**DOI:** 10.1523/ENEURO.0137-24.2024

**Published:** 2024-07-09

**Authors:** Phillip L. W. Colmers, Muhammad Nauman Arshad, Jayanta Mukherjee, Shinghong Lin, Shu Fun Josephine Ng, Patrick Sarmiere, Paul A. Davies, Stephen J. Moss

**Affiliations:** ^1^Department of Neuroscience, Tufts University School of Medicine, Boston, Massachusetts 02111; ^2^Ovid Therapeutics, New York, New York 10001; ^3^Department of Neuroscience, Physiology and Pharmacology, University College London, London WC1 6BT, United Kingdom

**Keywords:** EEG, GABA, GABA-AT, OV329, seizure, tonic inhibition

## Abstract

γ-Aminobutyric acid (GABA) is the principal inhibitory neurotransmitter in the adult brain which mediates its rapid effects on neuronal excitability via ionotropic GABA_A_ receptors. GABA levels in the brain are critically dependent upon GABA-aminotransferase (GABA-AT) which promotes its degradation. Vigabatrin, a low-affinity GABA-AT inhibitor, exhibits anticonvulsant efficacy, but its use is limited due to cumulative ocular toxicity. OV329 is a rationally designed, next-generation GABA-AT inhibitor with enhanced potency. We demonstrate that sustained exposure to OV329 in mice reduces GABA-AT activity and subsequently elevates GABA levels in the brain. Parallel increases in the efficacy of GABAergic inhibition were evident, together with elevations in electroencephalographic delta power. Consistent with this, OV329 exposure reduced the severity of status epilepticus and the development of benzodiazepine refractory seizures. Thus, OV329 may be of utility in treating seizure disorders and associated pathologies that result from neuronal hyperexcitability.

## Significance Statement

Enhancing inhibitory control over neurons to reduce excitability is a common strategy in treating seizure disorders. Here, we describe a novel compound, OV329, which acts on a common pathway to vigabatrin to increase inhibitory signaling following a low repeated dose paradigm. In vivo application of OV329 exhibited enhanced tonic γ-aminobutyric acid signaling in mice at the synaptic level in the hippocampus and at the network level reduced seizure severity and the development of benzodiazepine refractory seizures. This suggests OV329 may be of clinical use in the treatment of seizure disorders.

## Introduction

Ionotropic GABA_A_ receptors (GABA_A_Rs) are the primary mediators of inhibition in the central nervous system and shape neural function by constraining the excitability of neurons. These ligand-gated ion channels mediate Cl^−^-dependent neuronal hyperpolarization, the unitary events that underpin fast synaptic or phasic inhibitory neurotransmission and sustained or tonic inhibition (Brickley and Mody, 2012; Braat and Kooy, 2015). Consistent with their essential roles in limiting neuronal excitability, deficits in fast inhibitory neurotransmission result in epilepsy and associated pathologies arising from aberrant neuronal hyperexcitability (Brickley and Mody, 2012; Braat and Kooy, 2015).

The efficacy of GABAergic transmission is critically dependent upon the rates of synthesis and degradation of γ-aminobutyric acid (GABA). GABA is catabolized to succinic semialdehyde by GABA-aminotransferase (GABA-AT), an enzyme expressed at high levels by both neurons and glia. Accordingly, inhibitors of GABA-AT, such as vigabatrin (VGB), are used to treat infantile spasms and focal seizures with impaired awareness in adults (French, 1999; Wheless et al., 2007). However, the therapeutic use of VGB is severely limited due to retinal degeneration (Wild et al., 2009; Maguire et al., 2010; Yang et al., 2012; Foroozan, 2018). The mechanisms underlying this toxicity may result from the low potency of VGB and its irreversible effects on GABA-AT activity and possible off-target effects that include inhibition of GABA uptake mechanisms (Schousboe et al., 1986; Eckstein-Ludwig et al., 1999; Walters et al., 2019).

Recently, a rationally designed mechanism-based inactivator of GABA-AT, OV329, has been synthesized (Juncosa et al., 2018). This compound exhibits a marked increase (200–1,000-fold) in potency for GABA-AT compared with VGB (Juncosa et al., 2018; Shen et al., 2020; Feja et al., 2021). OV329 itself is an unreactive compound that undergoes activation when used as a substrate by the GABA-AT enzyme; the metabolite of OV329 inhibits enzymatic function by forming a noncovalent tight-binding complex with a residue key to the catalytic function of GABA-AT (Shen et al., 2020; Weerawarna et al., 2021). In this study, we examined the effects of sustained exposure of mice to OV329 on the efficacy of GABAergic inhibition, on the development of benzodiazepine refractory status epileptics (SE), and on basal electroencephalographic (EEG) power. Sustained exposure of mice to OV329 reduced GABA-AT activity, increased steady-state GABA levels in the brain, and induced elevations in phasic and tonic inhibition. These modifications paralleled a significant increase in EEG power selectively in the delta frequency. OV329 reduced the severity of kainate-induced SE and prevented the development of benzodiazepine refractory seizures. Collectively, our results demonstrate that OV329 acts on GABA-AT to increase GABA accumulation in the brain, elevate GABAergic inhibition, and exert potent anticonvulsant efficacy.

## Materials and Methods

### Animals and drug treatments

Five- to seven-week-old male C57Bl6/J mice (group housed and kept on regular day/night cycle) were weighed and labeled with tail markings for identification purposes and received up to 6 d of repeated i.p. injections with volumes of 0.01 ml/g of vehicle (pH 7.4 PBS) or 5 mg/kg OV329 (sustained exposure/repeated dose), or a single injection of 5 mg/kg OV329 at the same volume. Cohorts of cohoused mice received i.p. injections between 12:00 and 2:00 P.M. Injections were staggered so that individual mice within the same cohort finished their final scheduled injection on successive days. Upon completing their injection schedule, mice were killed for experiments the following day within a 24 h postfinal injection window.

### GABA-AT assay

GABA-AT assay kit was procured from Biomedical Research Service, SUNY Buffalo, catalog #E-134. The assay was done following the manufacturers’ protocol using a whole-brain homogenate. Briefly, net tissue weights were noted accurately after tissue collection, and the volume of 1× lysis buffer (1:3 ratio) was determined according to the tissue weight. A handheld tissue homogenizer was used to grind the tissue, and to avoid heating during this step, samples were kept on ice. One hour after homogenization, samples were centrifuged at 15,000× *g* for 20 min. Supernatant was collected and stored immediately at −80°C for the enzyme assay. Protein concentrations were determined using the BCA assay kit per manufacturer's recommendations (Thermo Fisher Scientific/Pierce, catalog #23225). The enzyme assay was carried out in a 96-well optical bottom, black plate (Thermo Fisher Scientific, catalog #265301) following the supplier's protocol. Each sample had control and reaction wells which contained either water or GABA-AT substrate. Twenty micrograms of protein was used for this assay, and each sample was run in duplicate. Upon addition of the assay buffer, plates were incubated at 37°C for 60 min. To protect from light, plates were covered with aluminum foil and incubated at 37°C for another 60 min. Absorbance at 492 nm was measured using a plate reader (GloMax Discover, Promega). For each sample, the optical density (OD) value of the control well was subtracted from the reaction well, and the Δ-OD values were used to calculate the enzyme activity. GABA-AT activity was presented as % control, as vehicle-treated samples were considered as 100% and OV329-treated samples were normalized to control.

### Western blotting

Western blotting was done following the standard procedure using the Invitrogen NuPAGE system. Twenty micrograms of total protein was loaded in each well and transferred to a PVDF membrane and probed with rabbit monoclonal GABA-AT (1 µg/ml, Abcam, catalog #ab216465) and b-actin (Cell Signaling Technology, catalog #4970, 1:2,000) antibodies (Hines et al., 2013; Nathanson et al., 2019; Lee et al., 2022). Membranes were probed with goat anti-rabbit secondary antibodies (Jackson ImmunoResearch) and subsequently developed with an enhanced chemiluminescence kit (Pierce, Thermo Fisher scientific). Images were captured using a Bio-Rad image analyzer and quantified using ImageJ/Fiji software.

### GABA measurement by liquid chromatography/mass spectrometry (LC/MS/MS)

GABA concentration was measured using whole-brain homogenates prepared for the GABA-AT activity assay. Twenty-five microliters of tissue homogenate was treated with 300 μl of acetonitrile. After centrifugation, the supernatant was completely dried under nitrogen and reconstituted with 200 µl of water prior to LC/MS/MS detection. All samples were then analyzed with an API7500 equipped with Phenomenex C6-Phenyl HPLC column. For internal standard, nonlabeled GABA (Sigma-Aldrich: 43811) and isotopic GABA, d6-GABA (Sigma-Aldrich: 615587) were used. GABA was detected using a LC/MS/MS, Sciex API 5500 with an electrospray ionization (ESI) interface (fee for service, Harvard Taplin Facility). A GABA calibration curve was prepared from 1.56 to 200 µg/ml. In the mobile phase of HPLC, acetonitrile was used in the organic phase, and the aqueous phase was constituted with 0.02% formic acid and 2.5 mM ammonium formate in water. For the MS detection, ESI-positive mode was used. Transition ions for GABA were Q1 m/z 104.1 and Q3 m/z 87. Transition ions for GABA-d6 were Q1 m/z 110.1 and Q3 m/z 93.

### Slice preparation

Six- to eight-week-old male C57Bl6/J mice that received either 1 or 6 d of vehicle or OV329 injections were anesthetized with isoflurane before decapitation. The brain was rapidly extracted and placed in an ice-cold, oxygenated (5% CO_2_/95%O_2_) slicing solution containing the following (in mM): 126 NaCl, 2.5 KCl, 1.25 NaH_2_PO_4_, 2 MgCl_2_, 0.5 CaCl_2_, 10 glucose, and 26 NaHCO_3_, adjusted to pH 7.4 by NaOH and osmolarity 305–315 mmol/kg and allowed to cool before being blocked and glued to a vibratome stage. In an ice-cold bubbled slicing solution, coronal (350 μm) sections containing the hippocampus were prepared with a vibratome (Leica VT1200). Sections were then hemisected through the midline, then allowed to recover in an oxygenated aCSF at 32°C in an incubator for 1 h, and then kept bubbled at room temperature.

### Patch recordings

The recording chamber was perfused with an oxygenated aCSF containing the following (in mM): 126 NaCl, 2.5 KCl, 1.25 NaH_2_PO_4_, 2 MgCl_2_, 0.5 CaCl_2_, 1 glutamine, 1.5 Na pyruvate, 10 glucose, 26 NaHCO_3_, and 3 kynurenic acid and the GABA_B_R antagonist CGP54626 (1 μM in DMSO; CGP), adjusted to pH 7.4 by NaOH and osmolarity 305–315 mmol/kg; this solution was heated to 32°C via an inline heater (Warner Instruments). Cells were visualized with differential interference contrast optics, and whole-cell patch recordings were made using borosilicate glass pipettes (3–5 MΩ, World Precision Instruments) containing the following CsCl internal solution (in mM): 140 CsCl, 4 NaCl, 1 MgCl_2_, 0.1 EGTA, 10 HEPES, 2 Mg-ATP, and 0.3 Na_2_-GTP, adjusted to pH 7.25 by CsOH and osmolarity 290 mmol/kg. Recordings were amplified (MultiClamp 700B, Molecular Devices), digitized at 10 kHz (1440A digitizer, Molecular Devices), low-pass filtered at 2 kHz, and recorded (pClamp 10.7, Molecular Devices) for offline analysis (Clampfit 10.7, Molecular Devices; Mini Analysis, Synaptosoft). Access resistance was monitored throughout the recordings, and cells with an access resistance larger than 20 MΩ or a change greater than 20% were excluded from analysis.

Dentate gyrus granule cells (DGGCs) were voltage clamped to −60 mV and gap-free spontaneous inhibitory postsynaptic current (sIPSC) recordings of 5–8 min duration at baseline. The perfusate was then switched to aCSF containing the GABA_A_R antagonist picrotoxin (PTX; 100 µM). PTX was applied for 4–8 min until both phasic and tonic GABA_A_R-mediated activities in the slice had ceased, and the holding current had reached steady state (Colmers and Bains, 2018; Nathanson et al., 2019; Lee et al., 2022).

### Patch analysis

Spontaneous IPSCs recorded under all relevant conditions were analyzed with automated parameters in Mini Analysis (Synaptosoft) and confirmed manually (parameters: threshold, 20 pA; period to search a local maximum, 10,000 ms; time before a peak for baseline, 5,000 ms; period to search a decay time, 20,000 ms; fraction of peak to find a decay time, 0.37; period to average a baseline, 1,000 ms; area threshold, 10; number of points to average, 3; direction of peak, negative). The first 200 events observed, starting 120 s after recordings began, were analyzed for amplitude and interevent interval (IEI); cumulative distributions of both measures were tested for significant differences using via the Kolmogorov–Smirnov (KS) statistics. The decay kinetics of sIPSCs were estimated by calculating the weighted time constant based on a single or double exponential fit as described (Nathanson et al., 2019).

Tonic current shifts were analyzed in ClampFit; 30 s regions taken from stable baseline and PTX treatment conditions were selected and used to generate all-points histograms, which were fit with Gaussian curves to determine the peak value of the holding current (Colmers and Bains, 2018; Nathanson et al., 2019). The shift from baseline for their parameters was calculated for individual cells, and the mean difference between vehicle- and OV329-treated groups was determined using these values, normalized to cell capacitance, to calculate current density. To visualize the shift in the holding current, the all-points histograms were plotted normalized to baseline, with the PTX-induced shift centered at their group's mean to show differences in noise distribution in addition to the overall shift in the holding current. Data were entered into Prism (GraphPad Software) for data manipulation, normality testing, statistical analysis, and graphing.

### EEG surgeries and recording

Adult male C57Bl/6J mice (11 weeks old) were surgically implanted with head mounts to permit EEG recordings. Mice were anesthetized via isoflurane inhalation, and stereotaxically positioned three-channel EEG/electromyography (EMG) head mounts (Pinnacle Technology, catalog #8201) were superglued to the skull in alignment with lambda. For cortical recordings, four screws were placed in the skull above the frontal and parietal lobes; silver epoxy was applied to each screw head to improve electrical connectivity between the electrodes and the head mount. After the surgery, the mice recovered for 7 d in their home cages before experimentation. On the day of recording, mice were connected to the preamps in the recording chambers for an hour to acclimatize them to the chamber prior to any recording. Data were recorded with a three-channel system (Pinnacle Technology) using LabChart. For the sustained dose experiment, a 4-h-long baseline recording of freely behaving mice was obtained. Following this, mice were injected with 0.5 mg/kg OV329 (i.p.) for 6 consecutive days. Twenty-four hours after the sixth, and final, dose of OV329, mice received 20 mg/kg kainate (i.p.), and 2 h after the dose of kainate, mice received a single dose of 5 mg/kg diazepam (DZ; i.p.). Recordings were terminated 3 h after the kainite injection to study the effectiveness of OV329 pretreatment in terminating kainate-induced seizures. EEG recordings were sampled at 2 kHz, and a 1 V range was used throughout the experiment (Moore et al., 2018; Jarvis et al., 2023).

### EEG analysis

To evaluate the potential impact of a sustained dose of OV329 on baseline EEG power, a 10 min silent period was analyzed where there was no muscular movement detected in the EMG channel. This represented the pretreatment measurement. All mice were administered a dose of 0.5 mg/kg OV329 for 6 consecutive days. The effects of OV329 were examined 1 h after the injections during Days 2, 4, and 6 for another 10 min silent epoch and statistically compared with the pretreatment baseline EEG epoch. To compare the EEG signals, they were converted into the frequency domain via the fast Fourier transform (FFT) resulting in power spectral plots using the LabChart software, with an 8 K FFT size, Hann (cosine-bell), and 87.5% window overlap parameters. The EEG frequency analysis was performed by binning the total signal into various frequencies such as delta (0–4 Hz), theta (4–8 Hz), alpha (8–13 Hz), beta (13–30 Hz), and gamma (30–100 Hz; Lee et al., 2022; Jarvis et al., 2023). The contribution of each frequency band to total power was then determined and compared between the treatment groups.

EEG recordings were further analyzed to determine the onset to first seizure and SE. Seizure activity was defined as EEG activity 2.5 times the standard deviation of the preceding 1 min of activity, which persisted for at least 20 s. SE was defined as seizure activity lasting longer than 30 min or continuous events that were separated by <30 s return to baseline. To examine the effectiveness of OV329 in preventing the development of DZ-resistant SE, we waited 20 min after administering DZ to allow the animals to return to SE and then compared the remaining 40-min-long EEG epochs between the two treatment groups. One mouse in the vehicle group and two mice in the OV329-treated group did not respond to kainate; these mice were excluded from comparisons evaluating the anticonvulsant efficacy of OV329. Statistical tests were performed using GraphPad Prism. Values are expressed as mean ± standard error of the mean (SEM). The Shapiro–Wilk test was performed to find normal distribution of the data sets. For all comparisons, parametric and nonparametric *t* tests were performed as appropriate.

## Results

### Sustained exposure of mice to OV329 inhibits GABA-AT activity and increases GABA accumulation in the brain

**Figure 1.**
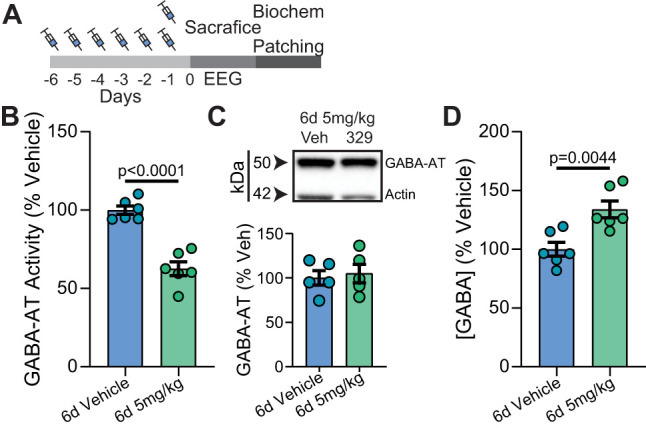
Examining the effects of OV329 on GABA-AT activity in vitro and in vivo. ***A***, The line diagram represents the i.p. dosing employed in our experiments. ***B***, GABA-AT activity in the brains of mice dosed with 5 mg/kg OV329 for 6 d was compared with vehicle-treated controls. ***C***, Brain extracts from mice dosed with OV329 were immunoblotted with GABA-AT and actin antibodies. The levels of GABA-AT in mice treated with OV329 were normalized to vehicle. ***D***, GABA levels were measured in the brains of mice treated with OV329 and values were normalized to vehicle. Data shown as mean ± SEM.

To examine the cumulative effects of OV329 on GABA-AT activity in the brain, mice were treated daily for 6 d with 5 mg/kg (i.p.; Fig. 1*A*). Sustained treatment with OV329 significantly reduced GABA-AT activity in the brain to 62.6 ± 4.4% of control (Fig. 1*B*; vehicle, 100.0 ± 2.7%; *n* = 6 mice per group; two-tailed unpaired *t* test). However, as measured by immunoblotting (Fig. 1*C*), OV329 did not modify the expression levels of GABA-AT (vehicle, 99.9 ± 8.2%; OV329, 105.0 ± 10.3%; *n* = 6 mice per group; two-tailed unpaired *t* test). Next, the effects of OV329 exposure on brain GABA levels were examined using liquid chromatography coupled with mass spectroscopy (Fig. 1*D*). OV329 treatment significantly increased GABA levels to 134.0 ± 7.2% of control (vehicle, 100.0 ± 5.9%; *n* = 6 mice per group; two-tailed unpaired *t* test). Thus, sustained exposure of mice to OV329 reduces GABA-AT activity without modifying its expression levels, which parallels increased GABA levels in the brain.

### Sustained exposure of mice to OV329 enhances tonic inhibition

To determine the functional effect of GABA-AT enzyme inhibition on inhibitory neurotransmission in the brain, electrophysiological recordings were taken following a 6 d drug injection paradigm. Mice (males, 6–8 weeks old at the time of experiment) received daily injections of either 6 d of vehicle, 6 d of 5 mg/kg OV329, or a single day of 5 mg/kg OV329 (Fig. 1*A*). About 24 h after the last injection, brain slices containing the hippocampus were prepared in which whole-cell patch-clamp recordings of DGGCs were held in voltage clamp at −60 mV (Modgil et al., 2017; Nathanson et al., 2019). We recorded from stable cells for 5–8 min and then applied the noncompetitive GABA_A_R antagonist PTX (100 µM) to the slice for 4–8 min. As expected, both the fast, phasic inhibition mediated by synaptic GABA_A_R and slow, tonic inhibition mediated by extrasynaptic GABA_A_R were blocked by PTX (Modgil et al., 2017; Nathanson et al., 2019).

**Figure 2.**
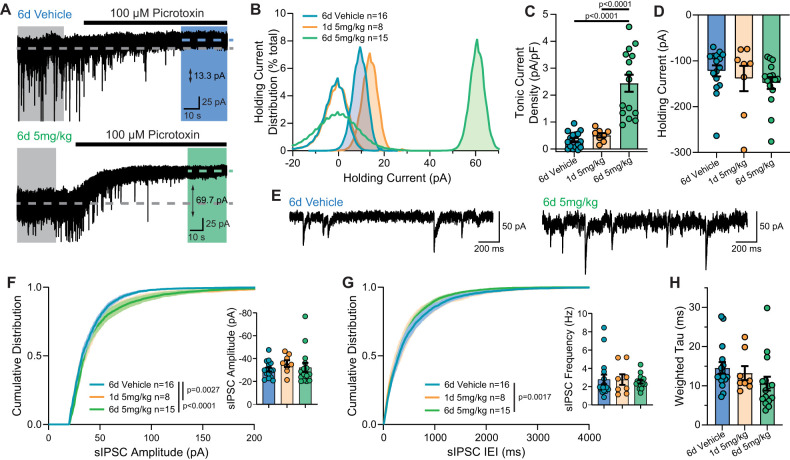
Repeated doses of 5 mg/kg OV329 significantly enhances tonic and phasic inhibition of DGGCs. Mice were given 6 d of repeated injections of vehicle (blue) or 5 mg/kg OV329 (green) or a single dose of 5 mg/kg OV329 (orange) prior to collection of brain slices for whole-cell patch-clamp recordings on DGGCs. Representative recordings in hippocampal slices of mice treated with 6 d repeated vehicle (***A***, top) or 5 mg/kg OV329 (***A***, bottom) showing a shift in the whole-cell holding current of DGGCs at baseline (gray-shaded region) following application of the noncompetitive GABA_A_R antagonist PTX (colored-shaded region) resulting from the blockade of extrasynaptic GABA_A_Rs. ***B***, All-points histogram showing the mean shift in holding current distribution normalized to baseline (unshaded color-coded traces) following blockade of GABA_A_Rs (shaded color-coded traces). ***C***, Tonic GABA current shift normalized to cell capacitance shows significant difference in current density following repeated dosing of OV329 at 5 mg/kg. ***D***, Despite differences in the magnitude of tonic current, the holding current at baseline was unchanged. ***E***, Representative spontaneous IPSC traces in mice given repeated doses of vehicle (left) or 5 mg/kg OV329 (right). Cumulative distribution of sIPSC amplitude (***F***) showing no significant differences in the mean amplitude (***F***, inset) following single or repeated doses of 5 mg/kg OV329. Cumulative distribution of sIPSC IEI (***G***) showing no significant differences in the mean frequency (***G***, inset) following single or repeated doses of 5 mg/kg OV329. ***H***, Weighted decay of sIPSCs showing no significant changes following repeated 5 mg/kg OV329 administration. Data shown as mean ± SEM.

The degree of tonic inhibition was calculated by examining the holding current of cells prior to and following PTX application (Fig. 2*A*). The basal level of tonic inhibition present in vehicle-treated mice (9.7 ± 2.2 pA; *n* = 16; *N* = 5 mice) was significantly enhanced in DGGCs from mice after 6 d of sustained treatment with 5 mg/kg OV329 (60.8 ± 2.2 pA; *n* = 15; *N* = 4 mice). However, a single dose of 5 mg/kg OV329 24 h prior was unable to significantly enhance the tonic inhibitory tone (13.2 ± 2.9 pA; *n* = 8; *N* = 5 mice; Fig. 2*B*; one-way repeated measures (RM) ANOVA: *F*_(2,36) _= 34.44). To control for the density of extrasynaptic GABA_A_R expression across cells, tonic current amplitudes were normalized to cell capacitance (Saliba et al., 2012; Fig. 2*C*; one-way RM ANOVA: *F*_(2,36) _= 30.7). Repeated dosing with 5 mg/kg OV329 significantly enhanced current density (2.4 ± 0.2 pA/pF; *n* = 15; *N* = 4 mice) compared with repeated doses with vehicle (0.3 ± 0.08 pA/pF; *n* = 16; *N* = 5 mice) or a single dose of 5 mg/kg OV329 (0.1 ± 0.08 pA/pF; *n* = 8; *N* = 5 mice). Despite the enhanced tonic current seen following sustained OV329 treatment, the basal holding current was unaltered across all conditions (Fig. 2*D*; vehicle: −121.9 ± 12.1 pA, *n* = 16; 6 d 5 mg/kg: −148.5 ± 13.0 pA, *n* = 15; 1 d 5 mg/kg: −38.7 ± 27.6 pA, *n* = 8; *N* = 4–5 mice per group; one-way RM ANOVA: *F*_(2,36) _= 0.89).

We next examined the effects of single and repeated daily doses of 5 mg/kg OV329 on phasic inhibition, in recordings of (action potential-dependent) sIPSCs from DGGCs held at −60 mV (Fig. 2*E*). The amplitude and duration between individual sIPSC events (IEI) were analyzed from the first 200 events recorded 2 min following the start of the recording, which allowed sufficient time for the pipette solution to equilibrate into the cell. Cumulative distribution plots were generated for the spontaneous amplitude (Fig. 2*F*) in which the distribution of amplitudes in both 1 d 5 mg/kg (*p* = 0.0027; KS test) and 6 d 5 mg/kg OV329 (*p* < 0.0001; KS test) show a significant right-shift compared with 6 d vehicle, consistent with a greater number of large events seen in DGGCs after OV329 treatment. Interestingly, however, the mean sIPSC amplitudes were not significantly changed by the drug (Fig. 2*F*, inset; 6 d vehicle: −30.5 ± 1.0 pA, *n* = 16; 1 d 5 mg/kg: −35.6 ± 2.9 pA, *n* = 8; 6 d 5 mg/kg: −32.2 ± 4.0 pA, *n* = 15; *N* = 4–5 mice per group; one-way RM ANOVA: *F*_(2,36) _= 0.55). Cumulative distribution plots generated for sIPSC IEIs (Fig. 2*H*) in mice treated with 5 mg/kg OV329 for 1 d (*p* = 0.0502; KS test) or 6 d (*p* = 0.0017; KS test) were left-shifted relative to those from 6 d vehicle-treated mice, consistent with a shorter duration between sIPSC events. However, the mean sIPSC frequency was not significantly altered (Fig. 2*G*, inset; 6 d vehicle: 2.8 ± 0.5 Hz, *n* = 16; 6 d 5 mg/kg: 2.5 ± 0.2 Hz, *n* = 15, 1 d 5 mg/kg: 2.8 ± 0.6 Hz, *n* = 8; *N* = 4–5 mice per group; one-way RM ANOVA: *F*_(2,36) _= 0.11). The mean decay kinetics of sIPSC decay (Fig. 2*J*) were also not significantly altered as measured by the weighted time constant (*τ_w_*) in response to single or repeated doses of 5 mg/kg OV329 compared with vehicle (*τ_w_* vehicle: 14.6 ± 1.5, *n* = 16 ms; *τ_w_* 6 d 5 mg/kg: 10.5 ± 1.8 ms; *n* = 15; *τ_w_* 1 d 5 mg/kg: 13.2 ± 1.8 ms, *n* = 8; *N* = 4–5 mice per group; one-way RM ANOVA: *F*_(2,36) _= 1.70).

### Sustained exposure with 0.5 mg/kg OV329 enhances phasic and tonic inhibition

**Figure 3.**
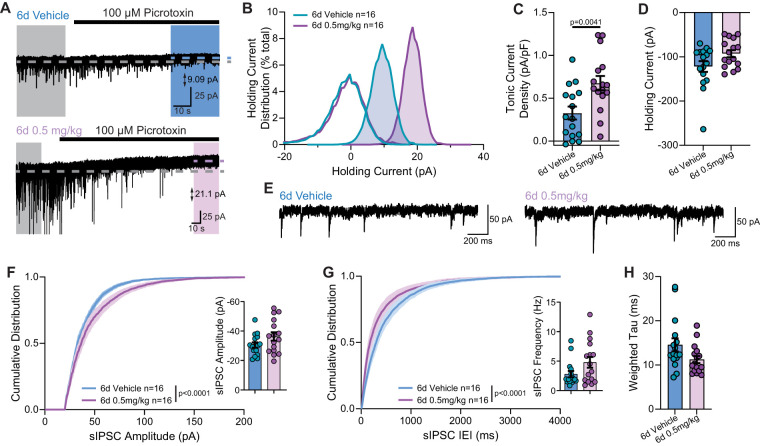
Repeated low doses of 0.5 mg/kg OV329 provide a significant enhancement to tonic and phasic inhibition onto DGGCs. Mice were given 6 d of repeated injections of vehicle (blue) or 0.5 mg/kg OV329 (purple) prior to experimentation. Representative recordings in slices from mice given 6 d repeated vehicle (***A***, top) or 0.5 mg/kg OV329 (***A***, bottom) showing a holding current shift from baseline (gray region) following PTX application (colored region). ***B***, All-points histogram showing the mean tonic current shift from baseline (unshaded traces) following PTX application (shaded traces). ***C***, Repeated dosing of OV329 at 0.5 mg/kg elicited a significant shift in the tonic current density with no significant change in the holding current (***D***). ***E***, Representative spontaneous IPSC traces in mice given repeated doses of vehicle (left) or 0.5 mg/kg OV329 (right). Cumulative distribution of the sIPSC amplitude (***F***) showing no significant change in the mean amplitude (***F***, inset) in mice given repeated doses of 0.5 mg/kg OV329. Cumulative distribution of sIPSC IEI (***G***) with no significant change in the mean frequency (***G***, inset) following repeated doses of 0.5 mg/kg OV329. ***H***, Weighted decay of sIPSCs showing no significant change following repeated 0.5 mg/kg OV329 administration. Data shown as mean ± SEM.

We next studied the effects of repeated treatment with a lower dose of OV329 on GABA-AT function. After a 6 d injection protocol with 0.5 mg/kg OV329 (Fig. 1*A*), we recorded tonic (Fig. 3*A*) and phasic (Fig. 3*E*) GABA currents in DGCCs held at −60 mV. In these animals, the PTX-mediated blockade of GABA_A_Rs unmasked a shift in the holding current (Fig. 3*B*; two-tailed unpaired *t* test: *p* = 0.018) in 0.5 mg/kg OV329-treated neurons (18.3 ± 2.7 pA; *n* = 16; *N* = 4 mice) significantly larger in OV329-treated than in vehicle-treated DGGCs (9.7 ± 2.2 pA; *n* = 16; *N* = 5 mice). Normalized to cell capacitance, the current density in mice given 6 d of 0.5 mg/kg OV329 (0.7 ± 0.1 pA/pF; *n* = 16; *N* = 4 mice) was significantly larger than in mice treated with 6 d of vehicle injections (0.3 ± 0.1 pA/pF; *n* = 16; *N* = 5 mice; Fig. 3*C*; two-tailed unpaired *t* test: *p* = 0.0041).

Following 6 d of 0.5 mg/kg treatment, the holding current for DGGCs at −60 mV was not significantly altered (Fig. 3*D*; two tailed unpaired *t* test: *p* = 0.055) following 0.5 mg/kg treatment (−93.0 ± 7.9 pA; *n* = 16; *N* = 4 mice) relative to vehicle treatment (−121.9 ± 12.1 pA; *n* = 16; *N* = 5 mice). sIPSC cumulative amplitude distribution (Fig. 3*F*) exhibited a right-shift (*p* < 0.0001; KS test) following 6 d of 0.5 mg/kg OV329 injections, consistent with a larger number of high-amplitude events, but no significant change to mean sIPSC amplitudes was observed (Fig. 3*F*, inset; 6 d vehicle: −30.5 ± 1.8 pA, *n* = 16, *N* = 5; 6 d 0.5 mg/kg: −36.3 ± 2.8 pA, *n* = 16, *N* = 4; two-tailed unpaired *t* test: *p* = 0.093). Similarly, the cumulative distribution of sIPSC IEIs (Fig. 3*G*) exhibited a left-shift (*p* < 0.0001; KS test) in mice treated with 0.5 mg/kg OV329, consistent with more frequent sIPSC events; however, the elevation observed in the mean frequency values did not reach significance (Fig. 3*G*, inset; 6 d vehicle: 2.79 ± 0.5 Hz, *n* = 16, *N* = 5; 6 d 0.5 mg/kg: 4.8 ± 0.9 Hz, *n* = 16, *N* = 4; two-tailed unpaired *t* test: *p* = 0.067). The increase in sIPSC decay kinetics following repeated dosing with 0.5 mg/kg OV329 (Fig. 3*H*) did not reach statistical significance (*τ_w_* vehicle: 14.6 ± 1.5 ms, *n* = 16, *N* = 5; *τ_w_* 6 d 0.5 mg/kg: 11.2 ± 0.8 ms; *n* = 16, *N* = 4; two-tailed unpaired *t* test: *p* = 0.058).

### Sustained dosing of mice with 0.5 mg/kg OV329 selectively enhances EEG δ power

**Figure 4.**
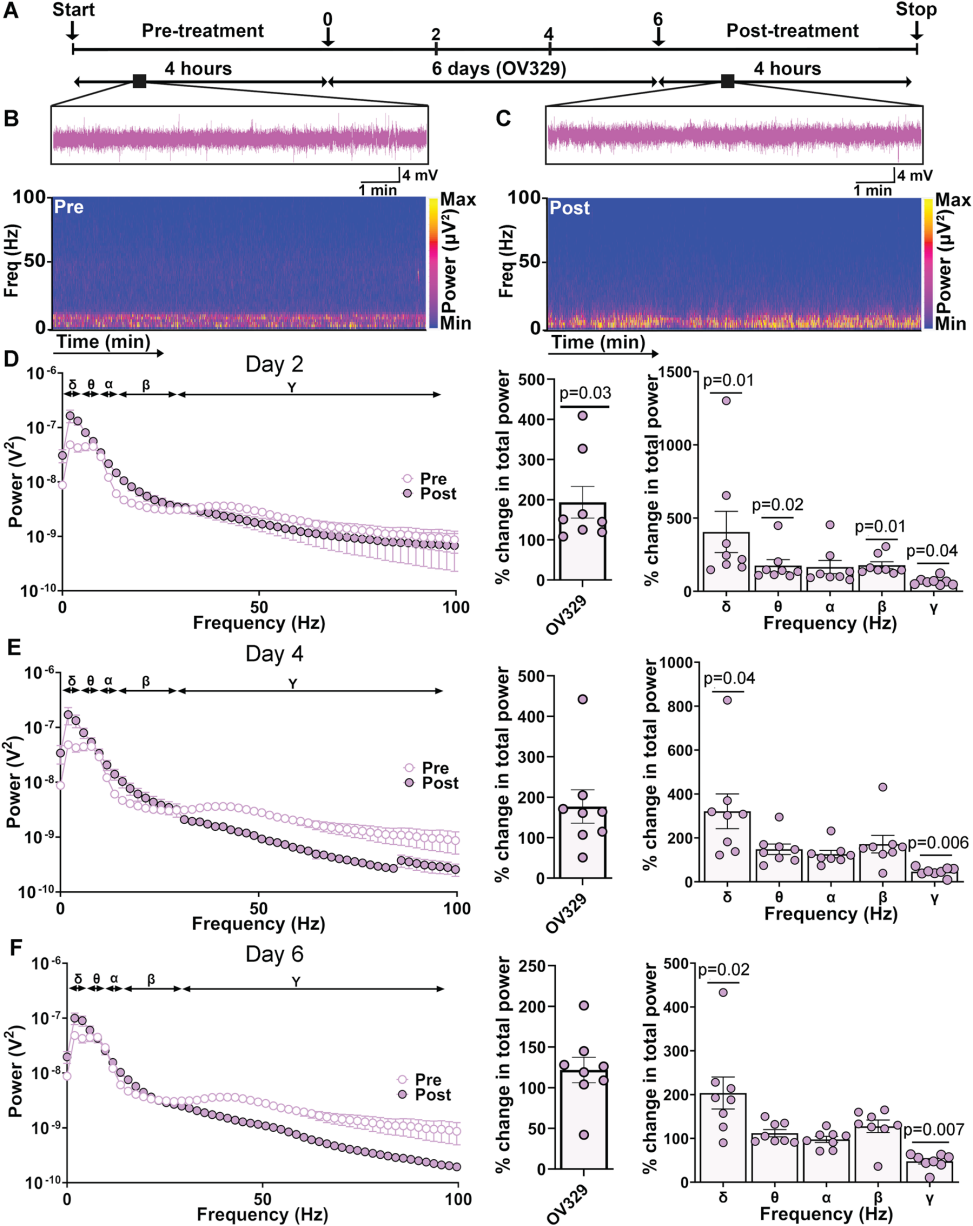
Sustained exposure to 0.5 mg/kg OV329 alters baseline EEG power. ***A***, Line diagram shows the experimental paradigm. ***B***, Representative EEG trace and its spectrogram show distribution of power across different frequency bands before OV329 treatment. ***C***, Representative EEG trace and its spectrogram show distribution of power across different frequency bands after treatment with 0.5 mg/kg OV329 for 6 consecutive days. ***D***, EEG recordings from Day 2 were subjected to FFT, and spectral plot is shown for frequencies between 0 and 100 Hz. The total EEG power was significantly increased following 2 d of OV329 treatment. Percentage change in EEG power was observed across delta, theta, beta, and gamma following 2 d of OV329 treatment. ***E***, EEG recordings from Day 4 were subjected to FFT, and spectral plot is shown for frequencies between 0 and 100 Hz. The total EEG power was not significantly elevated following 4 d of OV329 treatment; however, EEG power remained significantly altered across delta and gamma frequency bands. ***F***, EEG recordings from Day 6 were subjected to FFT, and spectral plot is shown for frequencies between 0 and 100 Hz. No change in the total EEG power following 6 d of OV329 treatment; EEG power remained significantly altered across delta and gamma frequency bands.

Administering a sustained dose of 0.5 mg/kg OV329 has been found to increase both tonic and phasic inhibition in the mice. We further examined whether this low sustained dose of OV329 had any impact on the baseline EEG in freely moving mice (Fig. 4*A*; Hines et al., 2013; Nathanson et al., 2019). A 4-h-long baseline recording was conducted (Fig. 4*B*), after which a single dose of 0.5 mg/kg was administered daily for 6 consecutive days, with recordings continuing for an additional 4 h after the final dose (Fig. 4*C*).

To study the effect of repeated doses of OV329 on EEG power, we compared 10 min segments during which there was no EMG activity from post-treatment timepoints on Days 2, 4, and 6 (Fig. 4*C*) against a pretreatment timepoint (Fig. 4*B*). To quantify the effects of OV329 on the EEG, recordings were converted into the frequency domain via FFT, generating a power spectral density plot for frequencies between 0 and 100 Hz (Fig. 4*D*, left; Nathanson et al., 2019; Jarvis et al., 2023). We normalized the post-treatment values in mice to their paired pretreatment values to calculate the percentage change and observed that OV329 significantly increased the total EEG power (Fig. 4*D*, middle; 193.5 ± 39.38% of pretreatment, two-tailed Wilcoxon matched-pairs signed-rank test (Wilcoxon *t* test): *p* = 0.03, *N* = 8). To assess possible effects on the EEG frequency bands (delta; 0–4 Hz, theta; 4–8 Hz, alpha; 8–13 Hz, beta; 13–30 Hz, and gamma; 30–100 Hz; Jarvis et al., 2023), we observed that OV329 treatment resulted in a significant power increase in the lower frequency domains, especially in the delta, theta, and beta power bands (Fig. 4*D*, right; delta: 405.0 ± 140.5%, *p* = 0.01; theta: 175.5 ± 40.55%, *p* = 0.02; beta: 177.9 ± 23.67%, *p* = 0.01; significance derived from Wilcoxon *t* test: *N* = 8). There was a concomitant decrease in the gamma power (Fig. 4*D*, right; 67.49 ± 9.07%, Wilcoxon *t* test: *p* = 0.04, *N* = 8 mice). On Day 4, power spectral density plots for frequencies between 0 and 100 Hz (Fig. 4*E*, left) showed no difference in the total EEG power (Fig. 4*E*, middle). However, we again observed a significant increase in the delta power band (Fig. 4*E*, right; 321.1 ± 79.21%, Wilcoxon *t* test: *p* = 0.04, *N* = 8) along with a reduction in the EEG power of the gamma frequency band (Fig. 4*E*, right; 46.38 ± 6.76%, Wilcoxon *t* test: *p* = 0.01, *N* = 8). By Day 6, spectral density analysis (Fig. 4*F*, left) again revealed no difference in the total EEG power (Fig. 4*F*, middle), but confirmed a persistent increase in the delta band power (Fig. 4*F*, right; 203.6 ± 36.5%, Wilcoxon *t* test: *p* = 0.02, *N* = 8) and the previous reduction in the gamma band power (Fig. 4*F*, right; 47.7 ± 6.2%, Wilcoxon *t* test: *p* = 0.007, *N* = 8).

### Sustained dosing of mice with OV329 reduces the severity of status epilepticus and prevents the development of benzodiazepine resistance

**Figure 5.**
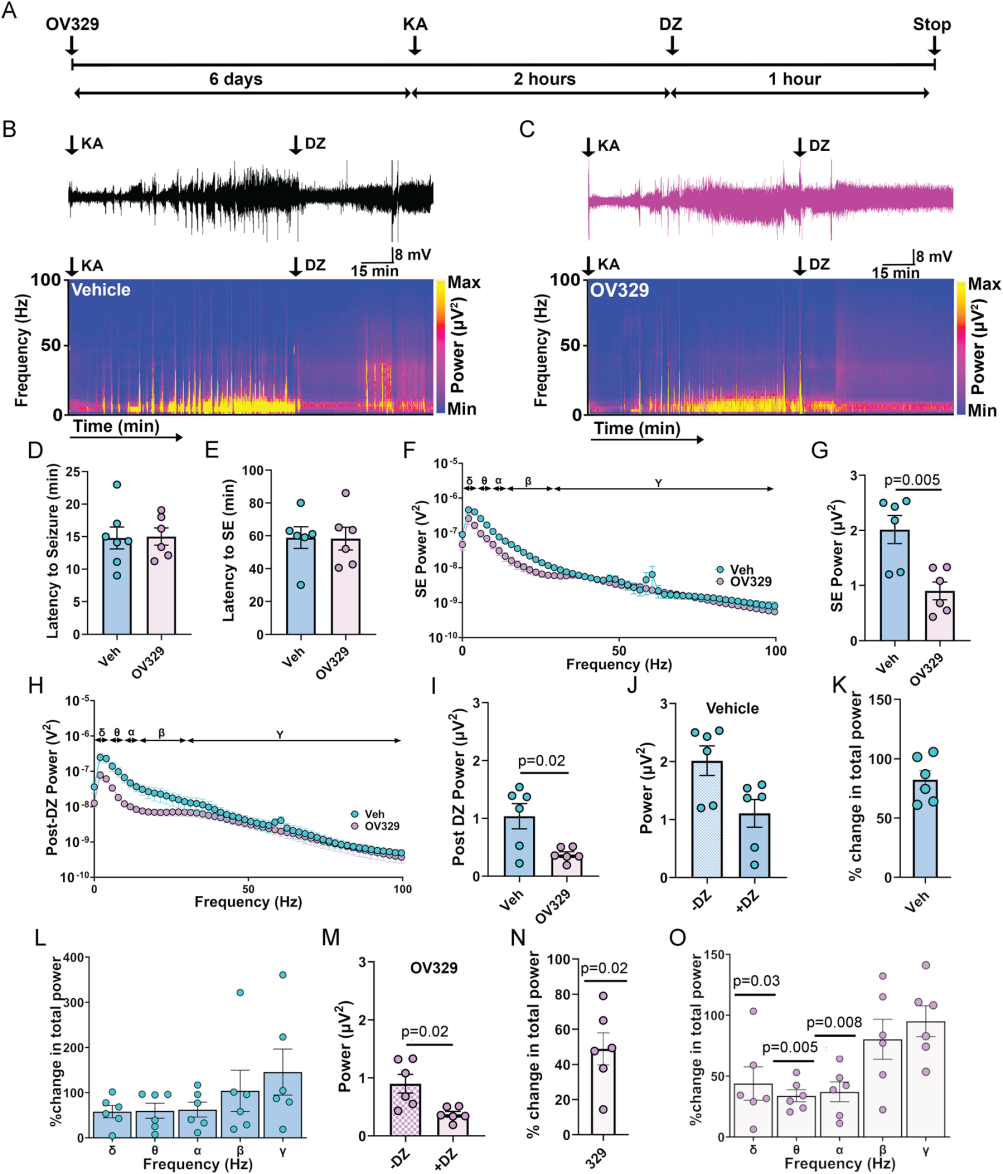
Sustained dosing with OV329 reduces the severity of SE. ***A***, Line diagram shows an experimental timeline we utilized. Sustained dosing of mice with 0.5 mg/kg OV329 enhances phasic and tonic inhibition in the DG. We further explored the effects of GABA-AT inhibition on the basal EEG power by performing subcortical EEG surgeries. Mice were implanted with EEG/EMG electrode to record a 4-h-long baseline (pretreatment period), and then mice received a single dose of 0.5 mg/kg OV329 for 6 consecutive days. Twenty-four hours after the final dose of OV329, mice received KA (20 mg/kg i.p.) to induce SE. Two hours following KA injection, mice were dosed i.p. with 5 mg/kg DZ, and EEG recordings were extended for a further 1 h. ***B***, Representative EEG trace and its spectrogram show distribution of power across different frequency bands for mice injected with vehicle (i.p.). ***C***, Representative EEG trace and its spectrogram show distribution of power across different frequency bands for mice injected with OV329. ***D***, Graph shows a comparison of latency to the first seizure between the two treatment groups. ***E***, A comparison of the onset of SE between the two treatment groups. ***F***, To provide the quantitative insights of effects of sustained dosage of OV329 on the basal EEG power, recordings were subjected to FFT, and EEG signal was converted from the time to the frequency domain to get power spectral density plot for frequencies between 0 and 100 Hz for both groups of mice experiencing SE. ***G***, Graph shows a comparison of SE power between the two treatment groups. ***H***, EEG recordings from mice pretreated with vehicle or OV329, 20 min following the DZ injection were subjected to FFT, and spectral plots are shown for frequencies between 0 and 100 Hz. ***I***, Graph shows a comparison of the total EEG power post DZ treatment between the two groups of mice. ***J***, Graph shows a comparison of EEG power before and after DZ treatment from the vehicle-treated mice. ***K***, Graph shows no difference in percentage change in the total EEG power after DZ treatment in vehicle-treated mice. ***L***, Vehicle treatment does not modify EEG power of any individual frequency band. ***M***, A comparison of EEG power before and after DZ treatment from OV329-treated mice. ***N***, Percentage change in the total EEG power was observed after DZ treatment in OV329-treated mice. ***O***, Percentage change in EEG power was observed across delta, theta, and alpha was observed upon OV329 treatment.

To explore the anticonvulsant properties of OV329, its effects on the development of kainic acid (KA)-induced SE in mice was examined using EEG recordings. This model was chosen because of the similarities with patients undergoing SE, as KA-induced seizures become refractory to DZ within minutes (Reddy and Kuruba, 2013; Lee et al., 2022; Vien et al., 2022; Jarvis et al., 2023). Mice implanted with EEG/EMG electrodes were injected with a single dose of 0.5 mg/kg OV329 for 6 consecutive days. Twenty-four hours after the final dose of OV329, mice received KA (20 mg/kg i.p.) to induce SE (Puttachary et al., 2015). EEG recordings captured the response of vehicle- and OV329-treated mice to KA injection for 2 h before a saturating concentration of DZ (5 mg/kg i.p.) was administered; recordings were terminated 1 h following DZ administration (Lee et al., 2022; Vien et al., 2022; Jarvis et al., 2023; Fig. 5*A–C*). The effects of OV329 on the development of SE were first examined, revealing no effects on latency to first seizure (Fig. 5*D*) or SE compared with vehicle (Fig. 5*E*).

In mice pretreated with OV329, the total EEG power of SE was significantly reduced compared with vehicle (Fig. 5*F,G*; vehicle, 2.0 ± 0.3 × 10^−6^ V^2^, and OV329, 0.9 ± 0.2 × 10^−6^ V^2^; two-tailed Welch's *t* test: *p* = 0.005; *N* = 6). Two hours after KA injection, mice were injected with DZ, and the ability of OV329 pretreatment to suppress EEG power was compared between the two groups 20 min after DZ injections. After DZ treatment, EEG power postictally was significantly reduced in OV329-treated mice compared with vehicle-treated mice (Fig. 5*H,I*; vehicle, 1.0 ± 0.2 × 10^−6^ V^2^, and OV329, 0.4 ± 0.04 × 10^−6^ V^2^; two-tailed Welch's *t* test: *p* = 0.02; *N* = 6). As in previous studies, DZ did not modify the total EEG power or any individual frequency band in mice pretreated with vehicle (Fig. 5*J–L*), as characterized by the resurgence in EEG power seen in Figure 5*C* (Goodkin et al., 2003; Silayeva et al., 2015; Bertoglio et al., 2017; Moore et al., 2018; Jarvis et al., 2023), consistent with the DZ insensitivity reported in the KA model (Reddy and Kuruba, 2013). By contrast, DZ significantly reduced EEG power in mice pretreated with OV329 (Fig. 5*M*; −DZ, 0.9 ± 0.2 × 10^−6^ V^2^, and +DZ, 0.4 ± 0.05 × 10^−6^ V^2^; Wilcoxon *t* test: *p* = 0.02, *N* = 6; Reddy and Kuruba, 2013; Lee et al., 2022; Vien et al., 2022; Jarvis et al., 2023). We found a significant reduction in the total EEG power following DZ treatment in mice pretreated with OV329 (Fig. 5*N*; 48.85 ± 9.10%; Wilcoxon *t* test: *p* = 0.03, *N* = 6). Examining specific frequency bands, this reduction in EEG power was observed across the delta (Fig. 5*O*; 43.95 ± 13.69%; Wilcoxon *t* test: *p* = 0.03, *N* = 6), theta (Fig. 5*O*; 33.91 ± 4.9%; Wilcoxon *t* test: *p* = 0.005, *N* = 6), and alpha (Fig. 5*O*; 37.14 ± 8.07%; Wilcoxon *t* test: *p* = 0.008, *N* = 6) frequency bands following OV329 pretreatment. Together, these findings suggest that increasing GABA-mediated inhibition with OV329 enhances the efficacy by which DZ terminates kainate-induced SE.

## Discussion

OV329 is a mechanism-based inactivator of GABA-AT, rationally designed for greater potency and selectivity (Juncosa et al., 2018; Feja et al., 2021) compared with VGB, an established GABA-AT mechanism-based inactivator with clinical application limited by its cumulative toxicity. Here we have examined the effects of sustained low dosing with OV329 in mice on the efficacy of GABA_A_R-mediated neuronal inhibition in the hippocampus. We found that 6 d of OV329 treatment at 5 mg/kg significantly enhanced tonic GABA_A_R-mediated current in DGGCs, while the mean sIPSC amplitude, decay, and frequency were not significantly changed. Consistent with this, we observed a significant elevation in brain GABA levels in treated mice that correlated with reductions in GABA-AT activity, without changes in its expression levels. Thus, the reduced GABA-AT activity and subsequent rise in brain GABA content are likely to underpin the increased tonic current measured in hippocampal slices following in vivo exposure to OV329. When mice were treated with just 0.5 mg/kg OV329 for 6 d, we also observed significant elevations in tonic inhibition—as with the higher dose. These results demonstrate that exposure of mice to OV329 increases the magnitude of neuronal inhibition mediated by extrasynaptic GABA_A_Rs, consistent with published studies showing VGB increases tonic inhibition in vitro (Jackson et al., 1994; Engel et al., 2001; Overstreet and Westbrook, 2001; Wu et al., 2001, 2003), but with greater potency (Wild et al., 2009; Maguire et al., 2010; Yang et al., 2012; Foroozan, 2018). The therapeutic effects of OV329 are time and dose dependent, as an acute dose of 40 mg/kg was able to reduce the severity of pentylenetetrazol-induced seizures and suppress generalized seizures in kindled rats, while an acute dose of 5 mg/kg had no effect (Feja et al., 2021). Accordingly, we show that an acute dose of 5 mg/kg is unable to significantly alter the tonic current in DGGCs; however, with sustained doses at 0.5 or 5 mg/kg, the cumulative inhibition of GABA-AT over multiple days was able to significantly enhance tonic GABA signaling at lower doses. The mechanism for VGB-associated visual field loss is unclear; however, it is thought that the high daily dose, poor blood–brain barrier permeability, and low inactivation efficiency may play a role (Wild et al., 2009; Maguire et al., 2010; Yang et al., 2012; Foroozan, 2018; Shen et al., 2020). Recent evidence suggests a role for off-target or downstream alterations in neuroactive amino acids (Jammoul et al., 2009; Walters et al., 2019). CPP-115, a cyclopentane-based precursor of OV329 with similar structure, was tested for retinal damage in a model for infantile spasms in rats. CPP-115 was given at a dose roughly 20-fold its effective dose for 45 d and showed a substantially lower retinal loss compared with VGB at its effective dose over the same duration (Silverman, 2012). This suggests OV329 may have a similarly enhanced safety profile; however, further examination is necessary.

To examine if these modifications to inhibition impact brain activity, we measured their effects on EEG power. Six days of treatment with 0.5 mg/kg OV329 significantly increased the power of delta frequency (0–4 Hz) and a decrease in gamma frequency (30–100 Hz). Similar elevations in delta power are seen with lorazepam and zolpidem, benzodiazepines that enhance phasic inhibition via their efficacy as GABA_A_R-positive allosteric modulators (Christian et al., 2015). Moreover, delta frequency EEG power also increases upon treatment with GABA reuptake inhibitor-based antiepileptic drugs (Frohlich et al., 2023). This suggests that OV329 treatment increases inhibition by regulating delta oscillations, which may suppress seizures.

To evaluate the anticonvulsant efficacy of OV329, we tested the effects of 6 d exposure to 5 mg/kg OV329 on the development and severity of SE induced by KA using EEG recording. While OV329 did not modify the latency to SE, the power of KA-induced seizures was reduced, suggesting this compound exhibits anticonvulsant activity. In agreement with our results, a single 40 mg/kg dose of OV329 has been reported to reduce the severity of pentylenetetrazol-induced convulsions as measured on the Racine scale (Feja et al., 2021).

Significantly, pretreatment of mice with OV329 prevented the development of benzodiazepine resistance following entrance into KA-induced SE. In humans, as in rodents, SE (Reddy and Kuruba, 2013; Lee et al., 2022; Jarvis et al., 2023) becomes resistant to benzodiazepines resulting in increased mortality of this trauma and severe brain damage in surviving patients and significantly increases the probability of developing chronic epilepsy (Walker, 2018; Crawshaw and Cock, 2020). Sustained elevations in neuronal excitability led to profound modifications in the efficacy of synaptic inhibition which include reductions in GABA levels, internalization of GABA_A_Rs, and modifications in their subunit composition, in addition to increased neuronal Cl^−^ accumulation (Walker, 2018; Burman et al., 2019, 2022). While further studies are required to understand the mechanism underlying the effects of OV329, presumably its ability to enhance the efficacy of GABAergic inhibition is of significance in preventing the development of benzodiazepine refractory seizures in SE.

In summary, our results demonstrate that OV329 treatment of mice induces sustained elevations in the magnitude of GABAergic inhibition and selective elevations in EEG delta power. These sustained effects on neuronal excitability suggest that OV329 may be of use as an anticonvulsant and for treatment of disorders that result from aberrant elevations in neuronal excitability.

## Synthesis

Reviewing Editor: Harald Sontheimer, University of Virginia Decisions are customarily a result of the Reviewing Editor and the peer reviewers coming together and discussing their recommendations until a consensus is reached. When revisions are invited, a fact-based synthesis statement explaining their decision and outlining what is needed to prepare a revision will be listed below. The following reviewer(s) agreed to reveal their identity: NONE. Note: If this manuscript was transferred from JNeurosci and a decision was made to accept the manuscript without peer review, a brief statement to this effect will instead be what is listed below.

Overall, there was some enthusiasm about the identification and characterization of a new GABA transaminase inhibitor that closely mimics prior results obtained with vigabatrin. However the reviewers have concerns about the validity of the difference in safety between the new drugs and established drugs. Also one reviewer is concerned about the seizure data and incomplete description of results using diazepam in treated/untreated animals (i.e., rates of seizure recurrence, descriptions of effects on EEG power spectra). The full reviews are attached as there is too much detail to be combined into a consensus statement.

Referee 1:

This article describes effects of a new, high-affinity inhibitor of GABA transaminase (OV329) on GABA-A receptor currents and seizures. Similar to the established effects of the antiepileptic drug vigabatrin (another GABA transaminase inhibitor), OV 329 increased brain GABA levels, enhanced tonic GABA-A currents and affected acute seizures induced by kainic acid. The effects on acute, kainite-induced seizures were fairly modest, however, with no change in latencies to first seizure, status epilepticus, but descriptions of actual seizure severity/duration in vehicle- vs. OV329-treated animals is not described. There were EEG changes seen in OV329-treated mice (i.e., higher delta frequency power and reduced gamma frequency power interictally, lower total EEG power during seizures). Of greater interest is the finding that seizures/status in OV329-treated mice showed greater responsiveness to diazepam than vehicle-treated mice. This new molecule is of broad interest because vigabatrin is recognized as a highly-efficacious AED (even in refractory cases) but its use is limited because of risks of permanent retinal injury that require routine and periodic ophthalmologic evaluations during use of the drug. The data appear to be of high quality and support the authors conclusions.

I have several comments to offer.

Major comments:

1. Figure 2. Authors should consider separating histograms in panel B into individual graphs for each condition (or at least showing vehicle and 1 day treatment OV 329 and another panel showing histograms after 6d treatment).

2. Figure 2. Authors should consider labeling panel C as "Tonic GABA current density" to clearly indicate this is the picrotoxin-sensitive current. This is appropriate because right-side of panel C shows holding current; are the authors surprised that no change in holding current was seen after OV329 treatment? How do the authors explain an increase in PTX-sensitive tonic current without any changes in holding current? (Cl- shifts, alteration in other resting conductances seem unlikely given intracellular solutions that were designed to isolate GABA-A currents).

3. Figure 5 and results. The reader would benefit from additional descriptions about why the authors chose to simply illustrate and describe "Total EEG power" after statis epilepticus versus presenting power for individual frequency bands as done in prior figure.

4. Figure 5 results. Additional information about seizures would be helpful. For example, were there differences in seizure severity (racine class), mortality in OV329-treated animals? Did all animals in both groups develop status epilepticus?

5. Discussion, page25, line 542. It would be appropriate to cite work of MacDonald, Kapur, and Goodkin on the effects of status epilepticus on GABA-A receptor trafficking that contributes to benzodiazepine resistance. Upregulation of tonic GABA-A receptors/enhancement of tonic GABA currents may be protective but conventional benzodiazepines are believed to act on synaptic GABA-A receptors containing gamma2 subunits (that internalize during status). Do the authors believe that in presence of higher GABA concentrations BZDs are modifying tonic GABA currents or is it believed that OV329 somehow prevents internalization of BZD-sensitive receptors?

6. Did any OV329-treated animals experience recurrent seizures after diazepam therapy? Illustrating the % of animals in each group that showed recurrent seizures after diazepam would be helpful to readers.

7. The authors should include some mention of limitations of their acute seizure model in discussion and highlight that it remains to be shown how OV329 affects spontaneous seizures in chronic epilepsy models. It would also be appropriate to include discussions and comparisons of the increased brain GABA concentrations seen with OV329 to these described previously with vigabatrin (both MRI spectroscopy and microdialysis/HPLC measurements).

8. Finally, additional discussion of retinal toxicity should be included. If retinal toxicity were a direct consequence of GABA transaminase inhibition/increased GABA concentrations, OV329 may be expected to have the same effect as vigabatrin. Is vigabatrin retinal toxicity thought to be due to idiosyncratic effects? Are there any reasons to suggest that OV329 would be safer?

Referee 2:

OV329 had little effect on phasic inhibition at either dose (0.5 mg/kg and 5 mg/kg) given for 6 days, with only a slight shift in the cumulative distribution plots, but no change in mean sIPSC amplitude or frequency. In several places the authors describe these effects as showing that "phasic inhibition is increased" (lines 57, 344 &383) and other times they say that it is not changed (lines 279, 687). This ambivalence is warranted, because there is no evidence that the small shift in the cumulative distribution plots would have any functional effect, and the lack of effect on the mean amplitude of sIPSCs at both 0.5 and 5 mg/kg is more relevant. In contrast, there was a large and dose-dependent increase in tonic inhibition.

The authors compared their findings with OV329 to those of vigabatrin, another GABA-AT inhibitor, but their data from VGB is too superficial to make firm conclusions about any differences. In fact, on the face of it their results from OV329 are remarkably similar to those published for VGB (Wu et al, J Neurophysiol 89: 2021-2034, 2003), which causes an increase in tonic inhibition and a decrease in phasic inhibition. The authors did not see the decrease in sIPSC amplitude with OV329, but they did not examine a large enough range of doses of OV329 to know whether that would occur.

The authors state that OV329 is much more potent than VGB (saying it has a 200-1000 fold increase in "potency for GABA-AT compared to VGB"), and that VGB is a "low affinity GABA-AT inhibitor," but in doing so they ignore the difference in pharmacological mechanisms between the two drugs. VGB irreversibly inhibits GABA-AT, so that its effect is dependent on both concentration and duration of exposure, instead of just concentration (Gale and Iadarola, Science 208: 288-291, 1980). As a result, it has a sizeable effect on tonic inhibition at a concentration as low as 50 nM when given for 3-4 days (Wu et al, J Neurosci, 21(8):2630-2639, 2001). Therefore the authors protocol is comparing apples to oranges when they say (line 263) that VGB has an IC50 of 183.8 microMolar and OV329 has an IC50 of 104.1 nanoMolar. These values are both obtained after acute administration. Both drugs are used clinically with chronic dosing, so a single high dose of VGB is not relevant to its normal use. Although there are methodological differences between these studies (e.g. slices vs culture), a similar delayed effect (60 hours) occurred in vivo (Gale and Iadarola, 1980). Thus, unless the authors perform experiments to more fully characterize the dependence on dose and duration of OV329 and VGB, they can't claim that VGB is not as potent as OV329 when both are given for multiple days.

This becomes most relevant when the claim is made that OV329 "[may possibly avoid] the cumulative [visual] toxic effect seen with VGB." While there are reasons this may be true (e.g. visual effects of VGB may be due to off target effects, the irreversible mechanism, etc), the authors didn't perform any experiments to address this, so they should not speculate about such an important issue unless they have data that addresses it. On the other hand, the similarities and differences between the two drugs could offer an opportunity for future experiments to determine if OV329 is free of this side effect. If visual toxicity is due to some mechanism other than increased tonic inhibition then OV329 may not cause visual problems, but to make that claim will require data.

The authors describe the vehicle treated mice in Figure 5B as not being sensitive to treatment with diazepam, but the EEG trace and heat map in Figure B appear to show a large depression in power in response to diazepam. The lack of a significant effect in Figure 5J may be due to the experiment being underpowered.

## Author Response

Synthesis of Reviews:

Synthesis Statement for Author (Required):

Overall, there was enthusiasm about the identification and characterization of a new GABA transaminase inhibitor that closely mimics prior results obtained with vigabatrin. However the reviewers have concerns about the validity of the difference in safety between the new drugs and established drugs. Also one reviewer is concerned about the seizure data and incomplete description of results using diazepam in treated/untreated animals (i.e., rates of seizure recurrence, descriptions of effects on EEG power spectra). The full reviews are attached as there is too much detail to be combined into a consensus statement.

- Referee 1:

This article describes effects of a new, high-affinity inhibitor of GABA transaminase (OV329) on GABA-A receptor currents and seizures. Similar to the established effects of the antiepileptic drug vigabatrin (another GABA transaminase inhibitor), OV 329 increased brain GABA levels, enhanced tonic GABA-A currents and affected acute seizures induced by kainic acid. The effects on acute, kainite-induced seizures were fairly modest, however, with no change in latencies to first seizure, status epilepticus, but descriptions of actual seizure severity/duration in vehicle- vs. OV329-treated animals is not described. There were EEG changes seen in OV329-treated mice (i.e., higher delta frequency power and reduced gamma frequency power interictally, lower total EEG power during seizures). Of greater interest is the finding that seizures/status in OV329-treated mice showed greater responsiveness to diazepam than vehicle-treated mice. This new molecule is of broad interest because vigabatrin is recognized as a highly-efficacious AED (even in refractory cases) but its use is limited because of risks of permanent retinal injury that require routine and periodic ophthalmologic evaluations during use of the drug. The data appear to be of high quality and support the authors conclusions.

I have several comments to offer.

Major comments:

1. Figure 2. Authors should consider separating histograms in panel B into individual graphs for each condition (or at least showing vehicle and 1 day treatment OV 329 and another panel showing histograms after 6d treatment).

Response: These data were intentionally paired to illustrate that with the same dose of 5 mg/kg OV329 a single day is unable to significantly affect the tonic GABA currents while repeated doses act in concert to enhance tonic inhibition. Splitting the histograms into separate panels would take away from the magnitude of the shift that repeated doses elicits.

2. Figure 2. Authors should consider labeling panel C as "Tonic GABA current density" to clearly indicate this is the picrotoxin-sensitive current. This is appropriate because right-side of panel C shows holding current; are the authors surprised that no change in holding current was seen after OV329 treatment? How do the authors explain an increase in PTX-sensitive tonic current without any changes in holding current? (Cl- shifts, alteration in other resting conductances seem unlikely given intracellular solutions that were designed to isolate GABA-A currents).

Response: The figure is updated to reflect the suggested change. The lack of change in the holding current following repeated 5 mg/kg OV329 was surprising, however the variability in the baseline holding currents may have obscured the presence of an increased tonic GABA current following OV329 treatment.

3. Figure 5 and results. The reader would benefit from additional descriptions about why the authors chose to simply illustrate and describe "Total EEG power" after status epilepticus versus presenting power for individual frequency bands as done in prior figure.

Response: Thank you for this suggestion, these data have been incorporated into the updated figure.

4. Figure 5 results. Additional information about seizures would be helpful. For example, were there differences in seizure severity (racine class), mortality in OV329-treated animals? Did all animals in both groups develop status epilepticus? Response: The seizures were not video-monitored so Racine scale data were not collected. Mortality of the treatment was not determined, as the experiments were terminal. Not all animals developed status epilepticus in both groups, this information has been added to the methods section.

5. Discussion, page25, line 542. It would be appropriate to cite work of MacDonald, Kapur, and Goodkin on the effects of status epilepticus on GABA-A receptor trafficking that contributes to benzodiazepine resistance. Upregulation of tonic GABA-A receptors/enhancement of tonic GABA currents may be protective but conventional benzodiazepines are believed to act on synaptic GABA-A receptors containing gamma2 subunits (that internalize during status). Do the authors believe that in presence of higher GABA concentrations BZDs are modifying tonic GABA currents or is it believed that OV329 somehow prevents internalization of BZD-sensitive receptors? Response: We did not observe any significant changes in the kinetics of sIPSCs following repeated OV329 application that would lead us to suspect a change in the makeup of γ2-containing synaptic GABAARs. Follow up studies could include whole-cell patch clamp experiments in mice following a low magnesium challenge (Jarvis et al. 2023) to address this question.

6. Did any OV329-treated animals experience recurrent seizures after diazepam therapy? Illustrating the % of animals in each group that showed recurrent seizures after diazepam would be helpful to readers.

Response: The experiment ended without looking at recurrent seizures, thank you for the suggestion, we will incorporate this experiment into subsequent studies.

7. The authors should include some mention of limitations of their acute seizure model in discussion and highlight that it remains to be shown how OV329 affects spontaneous seizures in chronic epilepsy models. It would also be appropriate to include discussions and comparisons of the increased brain GABA concentrations seen with OV329 to these described previously with vigabatrin (both MRI spectroscopy and microdialysis/HPLC measurements).

Response: We have not looked at OV329 in the context of spontaneous seizures or a chronic epilepsy model. The findings in Figure 1C-E show repeated doses of OV329 suppress enzymatic activity of GABA-AT without affecting expression resulting in enhanced ambient GABA levels, however this was done without a dose response curve and comparing this to vigabatrin would be speculative.

8. Finally, additional discussion of retinal toxicity should be included. If retinal toxicity were a direct consequence of GABA transaminase inhibition/increased GABA concentrations, OV329 may be expected to have the same effect as vigabatrin. Is vigabatrin retinal toxicity thought to be due to idiosyncratic effects? Are there any reasons to suggest that OV329 would be safer? Response: Thank you, this suggestion has been incorporated into the discussion. - Referee 2:

OV329 had little effect on phasic inhibition at either dose (0.5 mg/kg and 5 mg/kg) given for 6 days, with only a slight shift in the cumulative distribution plots, but no change in mean sIPSC amplitude or frequency. In several places the authors describe these effects as showing that "phasic inhibition is increased" (lines 57, 344 &383) and other times they say that it is not changed (lines 279, 687). This ambivalence is warranted, because there is no evidence that the small shift in the cumulative distribution plots would have any functional effect, and the lack of effect on the mean amplitude of sIPSCs at both 0.5 and 5 mg/kg is more relevant. In contrast, there was a large and dose-dependent increase in tonic inhibition.

Response: Places in which the phasic inhibition was mentioned to be increased, we referred to the significant change in the distribution of sIPSC events. While the mean values were not significantly changed at either 0.5 or 5 mg/kg, the synergistic effect of increased distribution of faster and larger amplitude events at both doses is an exciting avenue for further study in addition to the striking change in tonic current.

The authors compared their findings with OV329 to those of vigabatrin, another GABA-AT inhibitor, but their data from VGB is too superficial to make firm conclusions about any differences. In fact, on the face of it their results from OV329 are remarkably similar to those published for VGB (Wu et al, J Neurophysiol 89: 2021-2034, 2003), which causes an increase in tonic inhibition and a decrease in phasic inhibition. The authors did not see the decrease in sIPSC amplitude with OV329, but they did not examine a large enough range of doses of OV329 to know whether that would occur.

Response: Our work describes for the first time use of a sustained low-dose paradigm of OV329 in vivo and its effects on GABAergic inhibition, EEG power and sensitivity to kainite induce seizures. We hope to address the potency of OV329 on sIPSC amplitude frequency and tonic inhibition in a future study.

The authors state that OV329 is much more potent than VGB (saying it has a 200-1000 fold increase in "potency for GABA-AT compared to VGB"), and that VGB is a "low affinity GABA-AT inhibitor," but in doing so they ignore the difference in pharmacological mechanisms between the two drugs. VGB irreversibly inhibits GABA-AT, so that its effect is dependent on both concentration and duration of exposure, instead of just concentration (Gale and Iadarola, Science 208: 288-291, 1980). As a result, it has a sizeable effect on tonic inhibition at a concentration as low as 50 nM when given for 3-4 days (Wu et al, J Neurosci, 21(8):2630-2639, 2001). Therefore the authors protocol is comparing apples to oranges when they say (line 263) that VGB has an IC50 of 183.8 microMolar and OV329 has an IC50 of 104.1 nanoMolar. These values are both obtained after acute administration. Both drugs are used clinically with chronic dosing, so a single high dose of VGB is not relevant to its normal use. Although there are methodological differences between these studies (e.g. slices vs culture), a similar delayed effect (60 hours) occurred in vivo (Gale and Iadarola, 1980). Thus, unless the authors perform experiments to more fully characterize the dependence on dose and duration of OV329 and VGB, they can't claim that VGB is not as potent as OV329 when both are given for multiple days.

Response: The higher potency of OV329 and the precursor compounds that preceded its development have been documented across several papers (Pan et al. 2003 PMID: 14640537; Juncosa et al. 2018; Shen et al. 2020). Further, OV329 is a mechanism based inactivator, whose ability to inhibit GABA-AT is also both concentration and time dependent. However, this experiment in particular may be better suited for another manuscript in which we examine the acute effects of both OV329 and VGB at different doses using a 1 hour incubation. Figure 1A has been removed from this manuscript.

This becomes most relevant when the claim is made that OV329 "[may possibly avoid] the cumulative [visual] toxic effect seen with VGB." While there are reasons this may be true (e.g. visual effects of VGB may be due to off target effects, the irreversible mechanism, etc), the authors didn't perform any experiments to address this, so they should not speculate about such an important issue unless they have data that addresses it. On the other hand, the similarities and differences between the two drugs could offer an opportunity for future experiments to determine if OV329 is free of this side effect. If visual toxicity is due to some mechanism other than increased tonic inhibition then OV329 may not cause visual problems, but to make that claim will require data.

Response: The wording of this has been altered in the discussion of the manuscript to address this point as well as Reviewer #1's comment #8.

The authors describe the vehicle treated mice in Figure 5B as not being sensitive to treatment with diazepam, but the EEG trace and heat map in Figure B appear to show a large depression in power in response to diazepam. The lack of a significant effect in Figure 5J may be due to the experiment being underpowered.

Response: We apologize for the confusion, the insensitivity to diazepam in Figure 5B is referring to a well-characterized resurgence in epileptic activity following BDZ-administration in status epilepticus (Goodkin et al., 2003; Liliya et al., 2015; Bertoglio et al., 2017; Moore et al., 2018; Jarvis et al., 2023). Here, this is visible in the power spectrum beginning approximately 20 minutes following diazepam administration in the vehicle treated mice showing increases in power up to the ~40 Hz range. This is absent in the power spectra from OV329 treated mice, where following diazepam administration the frequency distribution of power remains in the ~0-20 Hz range. This has been added to the methods and mentioned in the results of the manuscript.
